# FZUImageReg: A toolbox for medical image registration and dose fusion in cervical cancer radiotherapy

**DOI:** 10.1371/journal.pone.0174926

**Published:** 2017-04-07

**Authors:** Qinquan Gao, Shaohui Lin, Penggang Bai, Min Du, Xiaolei Ni, Dongzhong Ke, Tong Tong

**Affiliations:** 1 Fujian Provincial Key Lab of Medical Instrument & Pharmaceutical Technology, Fuzhou University, Fuzhou, Fujian Province, China; 2 Fujian Provincial Cancer Hospital, Fuzhou, Fujian Province, China; 3 First Hospital of Longyan City, Longyan, Fujian Province, China; 4 Athinoula A. Martinos Center for Biomedical Imaging, MGH/Harvard Medical School, Cambridge, Massachusetts, United States of America; North Shore Long Island Jewish Health System, UNITED STATES

## Abstract

The combination external-beam radiotherapy and high-dose-rate brachytherapy is a standard form of treatment for patients with locally advanced uterine cervical cancer. Personalized radiotherapy in cervical cancer requires efficient and accurate dose planning and assessment across these types of treatment. To achieve radiation dose assessment, accurate mapping of the dose distribution from HDR-BT onto EBRT is extremely important. However, few systems can achieve robust dose fusion and determine the accumulated dose distribution during the entire course of treatment. We have therefore developed a toolbox (FZUImageReg), which is a user-friendly dose fusion system based on hybrid image registration for radiation dose assessment in cervical cancer radiotherapy. The main part of the software consists of a collection of medical image registration algorithms and a modular design with a user-friendly interface, which allows users to quickly configure, test, monitor, and compare different registration methods for a specific application. Owing to the large deformation, the direct application of conventional state-of-the-art image registration methods is not sufficient for the accurate alignment of EBRT and HDR-BT images. To solve this problem, a multi-phase non-rigid registration method using local landmark-based free-form deformation is proposed for locally large deformation between EBRT and HDR-BT images, followed by intensity-based free-form deformation. With the transformation, the software also provides a dose mapping function according to the deformation field. The total dose distribution during the entire course of treatment can then be presented. Experimental results clearly show that the proposed system can achieve accurate registration between EBRT and HDR-BT images and provide radiation dose warping and fusion results for dose assessment in cervical cancer radiotherapy in terms of high accuracy and efficiency.

## 1. Introduction

Cervical cancer is the second most common malignant form of cancers in women worldwide [1]. Although the medical situation has improved, the incidence and mortality rate of cervical cancer are still increasing in parts of developing countries. Recent reports have shown that more cases of cervical cancer occur at a young age [2]. Radiation therapy, also referred to as radiotherapy, is a common form of treatment for cervical cancer [3]. In the modern era of accurate radiotherapy, many new radiotherapy techniques [4], such as adaptive radiotherapy and intensity-modulated radiotherapy, have been commonly used for the treatment of malignant cases of nasopharynx cancer, prostate cancer, and esophageal cancer [5–9]. Considering the distinctiveness of cervical tumors in their anatomical location, pathological type, and biological behaviors, high-dose-rate brachytherapy (HDR-BT) has been adopted as a boost to external-beam radiotherapy (EBRT) for localized cervical cancer [10]. However, in clinical practice these two methods are implemented individually, which results in multiple dose distributions associated with the corresponding anatomical image. It is extremely difficult for radiation oncologists to calculate the accumulated dose across multiple treatments [11–14], which may limit the advantages of the combination of EBRT and HDR-BT. Therefore, it is important for radiation oncologists to investigate a method that can accumulate the dose distribution across multiple treatments in cervical cancer as accurately as possible.

To improve the accuracy and outcome of radiotherapy, it is necessary to calculate the actual dose distribution over the entire course of treatment and to investigate the best path and dose for HDR-BT. The accumulated dose distribution from both EBRT and HDR-BT needs to be calculated accurately before the dose is assessed. To map the dose distributions, the registration of images captured during different treatments is an essential technique. However, accurate registration is still a challenge owing to organ and tissue deformations, patient weight loss, or tumor shrinkage during radiotherapy sessions. In particular, the brachytherapy applicator used in HDR-BT treatment induces complex anatomical deformations in the vagina and surrounding organs such as the bladder and rectum, and conventional registration methods struggle to deal with these large deformations in both shape and intensity. In most clinical situations, radiation oncologists carry out dose planning only in accordance with rigid transformations, owing to the limitations of technology in overcoming the large deformations in HDR-BT for cervical cancer. However, it is difficult to obtain the correct dose distribution by simply adding dose distributions together without taking organ deformations into account. Deformable image registration [15] is a crucial technique for the mapping of dose distribution from EBRT and HDR-BT, which plays an increasingly important role in modern radiotherapy [16–18]. To facilitate the assessment of radiation doses across EBRT and HDR-BT fractions, Christensen *et al*. [19] used a viscous fluid model to map EBRT images onto HDR-BT images. However, their approach was only evaluated over some organs of interest and their algorithm is based on the assumption that the restoring force is proportional to the velocity of the transformation. Berendsen *et al*. [20] proposed a geometric penalty based registration technique for EBRT and HDR-BT images, which folds the applicator region and bring the applicator volume down to zero. However, it relies on the assumption that an accurate model of the applicator is available, which is not always the case. Osorio *et al*. [21] registered several pairs of critical organs individually by segmentation and then combined all the individual registration results to obtain the final deformation field. However, the resulting deformation is not guaranteed to be diffeomorphic without constraining the individual transformation to be coherent. A similar approach has been suggested by Zhen *et al*. [22]. Recently, Kim *et al*. [23] attempted to transform the dose distribution from intensity-modulated radiotherapy into that from HDR-BT with magnetic resonance imaging using an intensity-based deformable registration method provided by a commercial software package (Velocity–AI 2.7). Six registrations out of 15 failed due to the complex anatomical deformations. A more robust and accurate method of medical image registration and a dose fusion system are required to deal with the complex anatomical deformations in EBRT and BT images for the assessment of cumulative doses throughout the course of multiple treatments.

Over recent decades, there have been a large amount of research devoted to the development of image registration technology [24–27]. The Insight Segmentation and Registration Toolkit [28] is the most popular open-source toolkit and also the most commonly used in the medical field. Elastix [29] is a toolbox that relies on the Insight Segmentation and Registration Toolkit and provides a broad range of rigid and non-rigid registration methods for medical images. The Images Registration Toolkit is another excellent toolkit for the medical image registration. It provides a collection of libraries and command–line tools for the processing and registration of medical image, including the most widely used non-rigid registration algorithm using free-form deformation (FFD) model based on B-spline [30]. Although the toolkits listed above can provide many excellent algorithms for the processing and registration of medical image, most of them unfortunately only provide generic tools for performing image processing and analysis, and rarely have a user-friendly interface and visualization functions. These are difficult for radiation oncologists to use for dose assessment across EBRT and HDR-BT fractions in cervical cancer. AMIDE [31] can provide a graphical interface, but only rigid algorithms are included. Although there are commercially available toolkits that have been widely used in clinical, the ‘unreasonable’ deformations and uneven brightness of voxels, which are induced by the brachytherapy applicator, associated with cervical cancer make image registration particularly challenging [32–33]. Therefore, it is important to develop a user-friendly system that provides easily integrated methods of image registration for complex tasks of image registration and enables accurate radiation dose fusion in radiotherapy.

In this paper, we have developed a toolbox for medical image registration and dose fusion, which is applied in the registration of EBRT and HDR-BT CT images and the mapping of radiotherapy doses in cervical cancer, providing a convenient and robust system for the assessment of radiotherapy doses. Several state-of-the-art registration methods are integrated into the proposed system, including rigid and affine registration, and non-rigid registration based on FFD using B-spline. In addition, to deal with large deformations in both shape and intensity that are present in radiotherapy images, a multi-phase non-rigid registration method using local landmark-based free-form deformation (LFFD) is proposed for locally large deformations between EBRT and HDR-BT CT images, followed by intensity-based FFD. The proposed system provides a user-friendly interface and real-time visualization, and simplifies the complex processes of the configuration of algorithm parameters. Dose accumulation and mapping functions are also provided. This will help in the assessment of the total dose distribution of multiple forms of radiation therapy in cervical cancer.

## 2. Materials and methods

The study complied with the principles expressed in the declaration of Helsinki and was approved by the Ethics Committee of Fujian Provincial Cancer Hospital. The need for informed consent was waived by the Ethics Committee because the study was an image processing and analysis. The images data that provided by Fujian Provincial Cancer Hosptial are anonymously, and without including any identification information of patients.

### 2.1 EBRT and HDR-BT image registration and dose fusion

**Fig 1:**
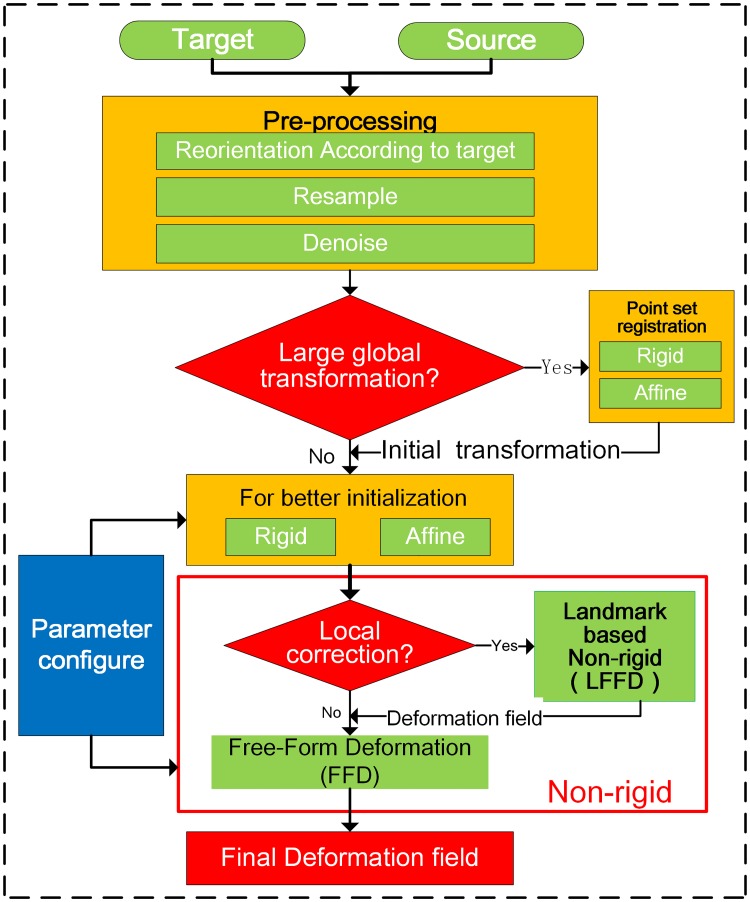
Flowchart and strategy of registration of EBRT and HDR-BT images in cervical cancer.

For dose assessment throughout the entire course of treatment, dose transformation is needed before dose accumulation [34]. Therefore, accurate registration between EBRT and HDR-BT images is particularly important. In this system, we propose a multi-phase hybrid registration strategy for the registration of EBRT and HDR-BT CT images in cervical cancer. Fig 1 shows the flowchart of the registration strategy.

In the first step, image pre-processing is carried out. The source (HDR-BT) image is reoriented according to the target (EBRT) image to give the same origin and orientation. After that, the source image is resampled according to the voxel spacing of the target image. Then, both target and source images are denoised with a median filter to attenuate the uneven brightness noise.

In the second step, global registration between EBRT and HDR-BT images is carried out for initial transformation. However, in some cases, there is a large global transformation, which causes direct intensity–based linear registration to fail. In these cases, point-based rigid or affine registration, which is also integrated into the system, can provide coarse registration. Landmarks are manually placed in both source and target images.

In the third step, the brachytherapy applicator and the bladder balloon, which are only present in the HDR-BT image, introduce structural dissimilarities between the EBRT and HDR-BT images. Complex anatomical deformations caused by the applicator and balloon compound the difficulties of registration. For more accurate transformation, we introduce a step of multi-phase non-rigid registration using LFFD, followed by intensity-based FFD. In the non-rigid registration process, many parameters need to be configured, such as the level of the registration pyramid, similarity metric, optimizer, interpolator, and so on. In particular, the control point spacing of the B-spline in the FFD model is the key component that will affect the registration performance. Configuration of all the parameters can easily be carried out via a user-friendly interface or parameter text files, which are provided by FZUImageReg.

**Fig 2:**
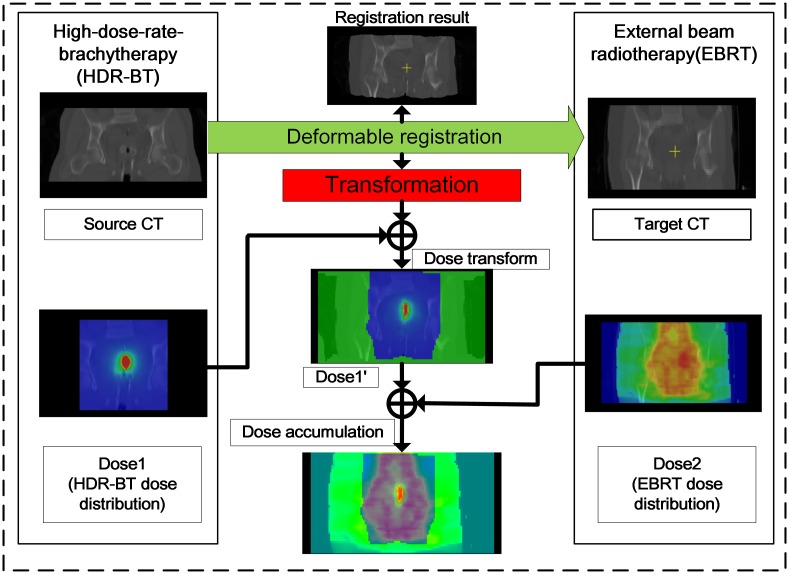
Scheme of dose accumulation between EBRT and HDR-BT.

After the registration process, we obtain the transformed HDR-BT image and the final transformation. This transformation describes how the HDR-BT image can map onto the EBRT image, which can be used to model the dose transformation between EBRT and HDR-BT for radiation dose accumulation [21]. Fig 2 shows the flowchart for creating dose accumulation via dose transformation.

Linear and deformable image registration is first performed between the target (EBRT) image and the source (HDR-BT) image to achieve an optimal transformation. The resulting transformation is then applied to the dose distribution map Dose1 in HDR-BT to create a transformed dose distribution map Dose1'. Finally, Dose1' and the dose distribution map Dose2 in EBRT can be summed to find the accumulated dose distribution.

In general, there are many radiotherapy fractions during an entire course of treatment in cervical cancer. Assuming that we have a series of *N* radiation computed tomography (CT) images *I*_*i*_ and dose distributions *D*_*i*_*(x)* in different treatment fractions, the role of deformable image registration is to generate the spatial transformations. The expression *T*_*iR*_*(x)* generates the position in the source image *I*_*i*_ that corresponds to position *x* in the reference image *I*_*R*_ The accumulated dose distribution can be defined as:

$$D_{Total}\left(x\right)=D_{R}\left(x\right)+\Sigma_{i=1,i\neq R}^{N}T_{iR}\left(D_{i}\left(x\right)\right)$$ (1)

where *D*_*Total*_*(x)* denotes the accumulated dose distribution; *D*_*R*_*(x)* denotes the reference, and *T*_*iR*_*(D*_*i*_*(x))* represents the *i*^th^ transformed dose distribution of the remaining *N*-1 treatment fractions.

### 2.2 System framework

**Fig 3:**
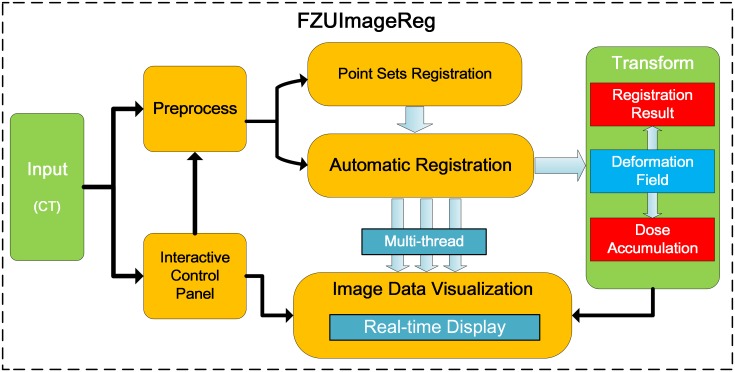
Framework and workflow of FZUImageReg system.

To achieve image registration and to determine the cumulative dose in an entire course of EBRT and HDR-BT in cervical cancer therapy, a toolbox for medical image registration and dose fusion with a user-friendly interface is proposed and implemented. This system was implemented in C++ on the basis of modular design technique. It consists of the following main functions as shown in Fig 3.

1. Pre-process: Medical image pre-processing techniques, including image denoising and resampling.
2. Point-based registration: For initial transformation in medical image registration with human input in cervical cancer therapy.
3. Image-based registration: For detailed transformation in medical image registration in cervical cancer therapy.
4. Visualization: Interactive display of 3D image data and 3D reconstructed model.
5. Dose fusion and visualization: Dose accumulation by warping the dose distribution in different treatments with the transformation calculated by registration.

The modular design of the system enables the user to design joint registration strategies and quickly configure, test, monitor and compare different registration methods for a specific registration application. In joint registration strategies, the output of one registration step is used as the input of the subsequent step and the final deformable transformations obtained as the result. With its modular design, the system can be used for most registration applications. Furthermore, multi-thread technology is used for multi-task parallel processing, including user interaction, medical image registration, and real-time visualization. The process of registration is carried out with a dynamic display, which allows users to monitor the process of registration and adjust the registration strategy accordingly to achieve an optimal result. With the transformation between EBRT and HDR-BT images, the accumulated dose can be easily calculated and visualized in our system.

In the following sections, we will introduce the core modules of this system. The strategy of registration of CT image in cervical cancer radiotherapy and the methods for dose accumulation of EBRT and HDR-BT are also demonstrated on the basis of this system.

#### 2.2.1 Image pre-processing module

**Fig 4:**
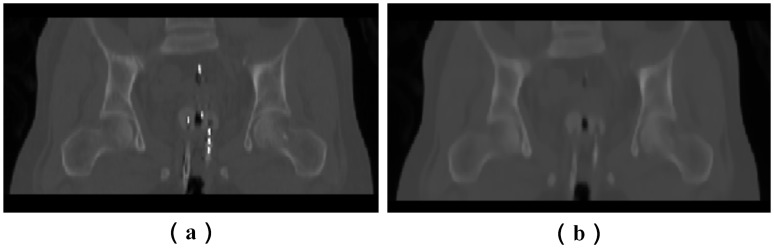
Denoising of HDR-BT images by median filter. (a) HDR-BT image with uneven bright voxels before denoising. (b) HDR-BT image after denoising.

CT image cervical cancer radiotherapy is easily affected by noise during their acquisition or transmission, which may affect their quality. In particular, the brachytherapy applicator, which is inserted into the patient’s vagina in HDR-BT treatment, will also introduce a large number of bright voxels in CT images, as shown in Fig 4(a). The registration accuracy of these CT images can be severely hampered by noise.

In order to remove unevenly distributed bright voxels, a median filter is introduced in this paper to attenuate noise without blurring the images. A median filter is a nonlinear digital filtering technique, which is widely used in digital image processing owing to its ability to preserve edges while removing noise. The HDR-BT image after denoising by a median filter is shown in Fig 4(b).

In addition, as EBRT and HDR-BT images are acquired separately, the representations of these images are quite different from each other, such as the image origin and orientation, voxel spacing, volume size, and so on. Therefore, it is preferable to convert these two types of images into a uniform representation before matching them. In this system, sampling of the source (HDR-BT) images according to the target (EBRT) ones is also supported by different interpolation techniques, including nearest neighbor, linear, C1-spline, B-spline and sinc interpolation.

#### 2.2.2 Image registration module

To achieve dose transformation and fusion in combined EBRT and HDR-BT treatments in cervical cancer, the key point is to align the images acquired in the different treatment processes as accurately as possible. In this system, we provide a powerful toolbox for medical image registration with a collection of rigid and non-rigid algorithms that are commonly used to solve different problems in medical image registration. In the optimization modules, several popular similarity metrics and optimization methods are provided to achieve an optimal transformation. Users are encouraged to design a personalized registration strategy according to specific tasks available in this platform.

The transformation model defines how the coordinates of two images are related. The following transformation models are currently supported in FZUImageReg: rigid, affine and non-rigid transformation. Rigid transformation is the simplest model, as it only requires translation and rotation, whereas affine transformation takes scaling and shearing further into consideration. However, neither rigid nor affine transformation is sufficient for the deformation of soft tissue. Therefore, we also introduce non-rigid registration using an FFD model based on B-spline, which was originally proposed by D.Rueckert *et*.*al*. [30], the main advantage of this model is that it enables automatic control of point placement on a regular grid, as well as local control.

In this model, transformation consists of two components: global transformation *T*_*global*_ and local deformation *T*_*local*_, which can be written as follows:

$$T\left(x,y,z\right)=T_{global}\left(x,y,z\right)+T_{local}\left(x,y,z\right)$$ (2)

where *T*_*global*_*(x*,*y*,*z)* is a global transformation that consists of rigid or affine transformation and *T*_*local*_*(x*,*y*,*z)* is a local deformation, that is represented by an FFD model based on B-Spline [30, 35].

Similarity metric describes the accuracy of the alignment of two images. Over recent decades, many similarity metrics have been proposed for measuring image alignment. In particular, voxel-based similarity measure using normalized mutual information (NMI) [36] has been shown to align multi-modality images with high accuracy and robustness. This method is based on the concept of information theory, which can be written as follows:

$$C_{similarity\left(NMI\right)}\left(A,B\right)=\frac{H\left(A\right)+H\left(B\right)}{H\left(A,B\right)}$$ (3)

where *H(A)* and *H(B)* denote the marginal entropies of *A* and *B* respectively and *H(A*,*B)* denotes their joint entropies. In addition to NMI, other commonly used metrics are also integrated into FZUImageReg, such as the sum of squares of intensity differences, cross correlation, mutual information, joint entropy and correlation ratio.

To maximize the similarity measure derived from the two images, the optimal transformation should be found. Therefore, the problem of registration can be formulated as a problem of optimization. The gradient descent method *∂C* / *∂Φ*_*i*,*j*,*k*_ is commonly used in optimization which does not rely on potentially insufficient statistics defined over small, local image regions. The following optimization methods are provided in FZUImageReg: gradient descent, steepest descent, downhill descent, conjugate gradient descent, SAM and SDM optimization [15]. Gradient descent is set as the default optimization method in the system.

#### 2.2.3 Large deformable registration

Large, local deformation is always present in the thigh region in CT images acquired by both EBRT and HDR-BT. This poses a significant challenge for fully automatic registration methods. In this paper, a multi-phase non-rigid registration method using LFFD is proposed for locally large deformation between EBRT and HDR-BT CT images, followed by intensity-based FFD. The local landmarks placed in the region of interest (ROI) are used for the correction of locally large deformation.

We assume that we have two sets of landmarks in the target image *P* = (*p*_*1*_, *p*_*2*_,⋯, *p*_*N*_) and the source image *Q* = (*q*_*1*_, *q*_*2*_,⋯, *q*_*N*_) respectively, where *N* is the number of landmarks. The landmarks in the point sets are the points of interest manually extracted from the thigh regions of CT images, as an example. The aim of this algorithm is to find an optimal transformation *T*_*local*_ for mapping EBRT and HDR-BT images together. The transformation *T*_*local*_ consists of two local transformations based on FFD using B-spline:

$$T_{local}\left(I\right)=T_{local}^{L=\left\{P,Q\right\}}\left(I\right)+T_{local}^{I}\left(I\right)$$ (4)

where $T_{local}^{L=\left\{P,Q\right\}}$ is an initial non-rigid transformation for the correction of locally large deformation. The movements of a landmark in ROI are given by a 3D tensor of three-dimension B-spline. In order to make the output of the initial transformation $T_{local}^{L=\left\{P,Q\right\}}$ to serve as the input of the subsequent transformation $T_{local}^{I}$ based on entire images, the control points of the FFD model are set to cover the entire image region in $T_{local}^{L=\left\{P,Q\right\}}$, and the control points that lie in the ROI are allowed to move during the landmark registration process, whereas those out of this region remain fixed. Assuming that Ω = {(*x*, *y*, *z*)|0 ≤ *x* ≤ *X*, 0 ≤ *y* ≤ *Y*, 0 ≤ *z* ≤ *Z*} is the entire image region and *ROI* ∈ *Ω* is the ROI. let *Φ* denote an *n*_*x*_ × *n*_*y*_ × *n*_*z*_ mesh of control points *φ*_*i*,*j*,*k*_ with a uniform spacing of *δ*. Then the transformation $T_{local}^{L=\left\{P,Q\right\}}$ can be written as follows:

$$T_{local}^{L=\left\{P,Q\right\}}\left(\text{I}\right)=T_{local}^{L=\left\{P,Q\right\}}\left(x,y,z\right)=\left\{\begin{array}{cc} \Sigma_{l=0}^{3}\Sigma_{m=0}^{3}\Sigma_{n=0}^{3}B_{l}\left(u\right)B_{m}\left(v\right)B_{n}\left(w\right)\phi_{i+l,j+m,k+n}, & \left(x,y,z\right)\in ROI\\ \left(x,y,z\right), & \left(\text{x},\text{y},\text{z}\right)\notin ROI\;\cap \left(x,y,z\right)\in \Omega \end{array}\right\}$$ (5)

where *i* = ⌊*x* / *n*_*x*_⌋ − 1, *j* = ⌊*y* / *n*_*y*_⌋ − 1, *k* = ⌊*z* / *n*_*z*_⌋ − 1, *u* = *x* / *n*_*x*_ − ⌊*x* / *n*_*x*_⌋, *v* = *y* / *n*_*y*_ − ⌊*y* / *n*_*y*_⌋, and *w* = *z* / *n*_*z*_ − ⌊*z* / *n*_*z*_⌋, and *B*_*l*_ is uniform B-spline basis function, which has previously been defined in [37].

To find the optimal transformation $T_{local}^{L=\left\{P,Q\right\}}$, we minimize the mean square of the Euclidean distance for each pair of landmarks in the transformed point sets $T_{local}^{L=\left\{P,Q\right\}}\left(P\right)$ and *Q*:

$$min\left\{D\left(T_{local}^{L=\left\{P\;,\;Q\right\}}\left(P\right),\;Q\right)=\Sigma_{P\in T_{local}^{L=\left\{P,Q\right\}}\left(P\right)}\Sigma_{Q}{\left(P-Q\right)}^{2}\right\}$$ (6)

**Fig 5:**
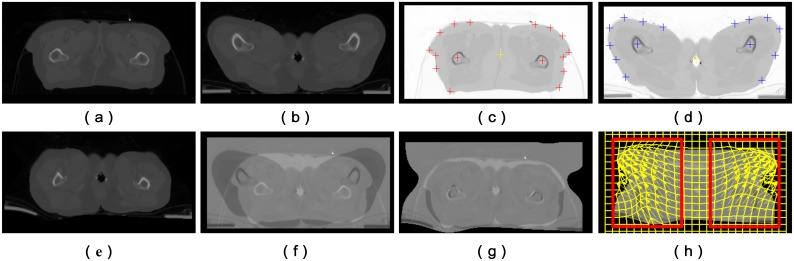
Correction of locally large deformation between EBRT and HDR-BT images by LFFD. (a) EBRT images used as a reference. (b) HDR-BT image with locally large deformation induced by the brachytherapy applicator. (c) and (d) Corresponding EBRT and HDR-BT image with manual landmarks. (e) HDR-BT image after correction of locally large deformation by LFFD. (f) and (g) Corresponding difference images before and after correction. (h) Deformation grid of LFFD, in which the ROIs are defined by the red rectangles.

With the optimal initial transformation $T_{local}^{L=\left\{P,Q\right\}}$ as an input, we then carried out intensity based non-rigid registration to obtain a further deformation $T_{local}^{I}$, which has previously been defined [30]. Fig 5 shows an example of the correction of large, local deformation correction in the thigh regions in cervical cancer radiotherapy images by the transformation $T_{local}^{L=\left\{P,Q\right\}}$.

#### 2.3.4 System characteristics and functions

**Fig 6:**
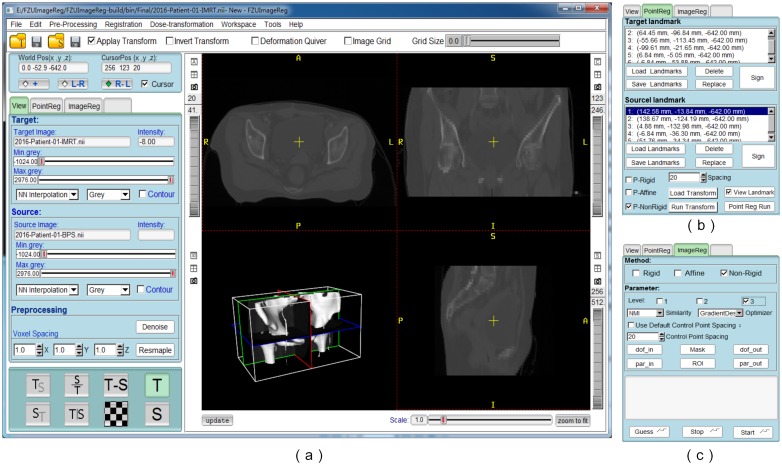
Screenshot of the proposed FZUImageReg system. (a) Main window interfaces of FZUImageReg (b) Point set registration panel (PointReg). (c) Automatic image registration panel (ImageReg).

The proposed system provides a user-friendly interface as shown in Fig 6(a). The right-hand widget is the visualization window, and the left-hand widget is the control panel of the system, including the menu bar, view panel, point-based registration panel for manually defined registration of landmarks, as shown in Fig 6(b). Intensity-based registration panel for automatic registration is shown in Fig 6(c).

The menu bar is used to load/save image data and image registration parameter files, and for dose transformation and other functions such as image preprocessing. For dose fusion, a file in DICOM-RT format for both EBRT and HDR-BT doses can be loaded into the system. The corresponding transformation generated by registration can also be added to create the transformed HDR-BT dose distribution by using the Dose-transformation button on the menu bar. After that, the accumulated dose distribution of the treatments can be calculated easily according to Eq (1). In addition, the dose distribution map before and after fusion can be displayed accordingly in the right-hand visualization widget, which is useful for the radiation oncologists to assess the dose distribution.

In the view panel, a set of image interaction functions are included, such as zooming, adjusting the grayscale of images, drawing contours by a thresholding method, and so on. A pseudo color display and different models of super position viewing of the target and source images are also supported. This can help users to easily understand and evaluate the differences in these two images before and after registration. In addition, image information, such as the intensity at the current cursor position can be shown in this panel.

Fig 6(b) show the point-based registration control panel-(PointReg). Using this panel, users can assign, delete, or replace landmarks directly over images for point set registration. Loading, and saving point sets from predefined files are also supported. These functions of point-based registration are rarely provided in other registration toolkits or software. In our system, rigid, affine, and non-rigid point set methods are all supported. The registration parameters and strategies can easily be configured for various point set registration applications.

The intensity-based registration panel (ImageReg) is shown in Fig 6(c). The registration algorithm includes rigid, affine, and the most widely used non-rigid registration methods based on FFD using B-splines. Several parameter configurations commonly used for registration method are directly provided in the panel. Additional parameters such as the number of iterations and the length of registration steps, can be configured via a parameter text file, which can be loaded onto the system using the par_in button. The ROI button defines an ROI for registration, and the dof_in button is used to load the initial transformation from previous steps for joint registration in hybrid registration method. This enables our system to be applied for most image registration problems. Compared to other image registration tools, our system provides a convenient interface for parameter configuration and simplifies the process of complex parameter setting for different tasks. This can easily be used by users who do not have professional registration skills.

## 3. Experiments and results

### 3.1 Data

In this paper, we applied the proposed system for CT image registration and dose accumulation in EBRT and HDR-BT treatment to 11 sets of clinical data from patients with locally advanced uterine cervical cancer. (The image data were anonymized and do not include any identifying information.) These sets of 3D volume data were provided by Fujian Provincial Cancer Hospital. All patients were treated by image-guided radiotherapy, including both EBRT and HDR-BT. The CT images were acquired with a Philips large aperture spiral CT scanner. The spatial resolution of all EBRT images is 0.9766 × 0.9766 × 5 mm, whereas the spatial resolution is 0.9766 × 0.9766 × 2.5 mm in the HDR-BT images. The scanning ranges in EBRT and HDR-BT are also different for patients and therefore result in differences in the size of 3D volume data.

**Fig 7:**
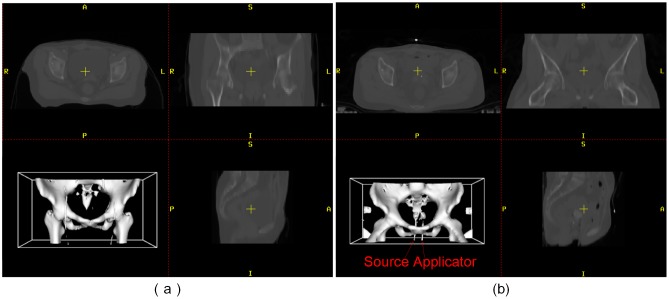
Original clinical data of one cervical cancer patient. (a) EBRT CT image. (b) HDR-BT CT image.

Fig 7 shows an example of EBRT and HDR-BT CT images of the same patient, and the corresponding 3D models of the pelvis are shown in the bottom left of the visualization windows. In this example, the EBRT images have a size of 512 × 246 × 40 voxels, the origin of the image is at (*x* = 0, *y* = −68.5, *z* = −642). Whereas the HDR-BT image have a size of 454 × 246 × 77 voxels, their origin is at (*x* = 3.094, *y* = −1016, *z* = −1458). These two images are not displayed in the same view window before pre-processing, owing to the differences in origin and orientation between the images.

In addition, the brachytherapy applicator is inserted into the patient’s body during an HDR-BT CT scan. This induces complex anatomical deformations in the uterus, bladder, and surrounding organs, and also many uneven bright voxels, as shown in Fig 4(a). To tackle this problem, image preprocessing methods mentioned in Section 2.2.1 were first applied.

In comparison to EBRT, the position of the thigh of the patient in HDR-BT treatment varied significantly owing to the use of the brachytherapy applicator, as shown in Fig 7(b). This “unreasonable” property present in EBRT and HDR-BT images poses a great challenge for conventional state-of-the-art registration methods.

### 3.2 Image registration and dose fusion

In the following experiments, we performed hybrid registration of EBRT and HDR-BT CT images according to the flowchart and strategy proposed in Section 2.1.

**Fig 8:**
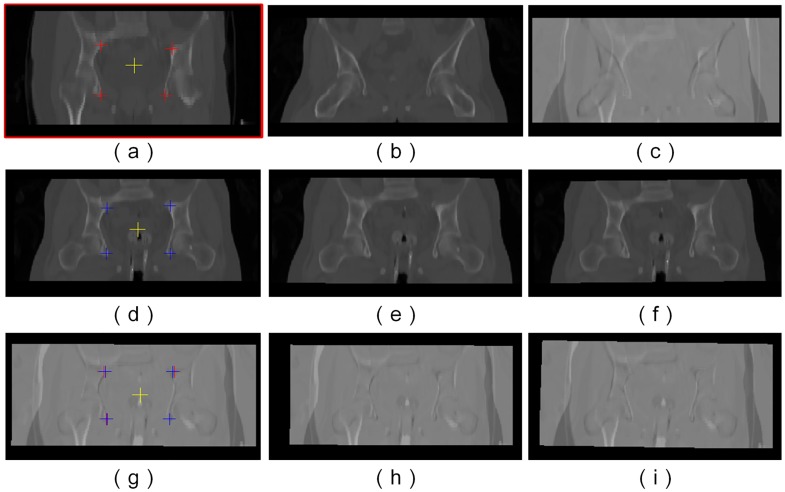
Coarse registration between EBRT and HDR-BT CT images for better initialization. (a) EBRT images used as a reference. (The four marked points inside the image were used for point set rigid registration.) (b) HDR-BT image before initialization. (c) Difference images between EBRT and HDR-BT before initialization. (d) HDR-BT image with four marked points used for point set rigid registration. (e) HDR-BT image after intensity-based rigid, and affine (f) registration. The corresponding difference images between EBRT and HDR-BT after point set rigid, intensity-based rigid, and affine registration are shown in (g)–(i).

There is a large, global transformation in the EBRT and HDR-BT images, which can be seen in Fig 8(c). Three coarse registration methods were applied for good initialization, namely point-based rigid, intensity-based rigid and affine registration. In point-based rigid registration, at least three pairs of landmarks are needed, which can be manually positioned directly using the PointReg panel. The selected landmarks should have the same anatomical structure. In this paper, we chose four landmarks, as shown in Fig 8(a) and 8(d).

The point set method works faster than intensity-based rigid or affine registration. However, the accuracy is affected by errors in the manually selected landmarks. After the initial point-based registration, there is still a considerable amount of misalignment between these two images. Therefore, the intensity-based rigid registration and affine registration were further carried out for a better alignment. The transformed HDR-BT images are shown in Fig 8(d)–8(f) and the corresponding difference images between the reference EBRT image and the transformed HDR-BT image are shown in Fig 8(g)–8(i).

With the initial transformation, we conducted two experiments to validate our proposed method for locally large deformation in EBRT and HDR-BT images. In the first experiment, only intensity-based non-rigid registration (FFD) was used directly, whereas in the second experiment, multi-phase non-rigid registration using LFFD was first carried out, followed by intensity-based FFD. For LFFD registration, the selected pair of landmarks has the same anatomical structure, which can be referred to Fig 5(e) and 5(f). We used a three-level deformation field with different hierarchical control point spacing. The initial control point spacing was set at 40 mm, since this provided the best results in our preliminary experiments, and was set to half this value at the next level. In both experiments, the same parameters were used for intensity-based non-rigid registration (FFD). We chose normalized mutual information as similarity metric, gradient descent for optimizer. Three levels of image resolution were adopted, and the initial control point spacing was set to 20 mm.

**Fig 9:**
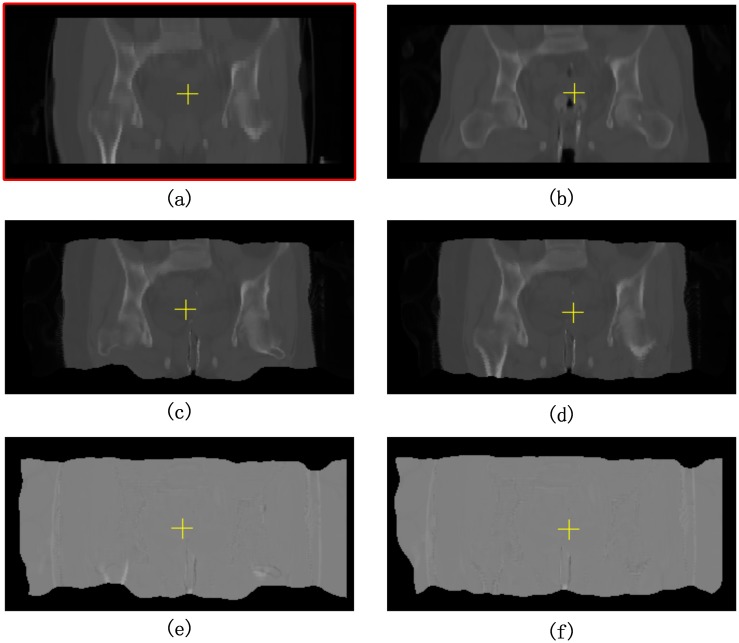
Comparison of registration results using FFD and LFFD+FFD non-rigid registration respectively. (a) EBRT image used as a reference. (b) Initialized HDR-BT image before non-rigid registration. (c) HDR-BT image after (FFD) non-rigid registration. (d) HDR-BT image after (LFFD+FFD) non-rigid registration. The corresponding difference images are shown in (e) and (f).

The results of the transformed source images after registration and the corresponding difference images from these two experiments are shown in Fig 9(c)–9(f). Compared to the initial affine registration in Fig 8(f) and 8(i), the results clearly show that the alignment of EBRT and HDR-BT images was improved significantly after FFD. Unfortunately, there is still a certain amount of misalignment in the thigh region, as shown in Fig 9(c) and 9(e). In other words, the anatomical alignment of locally large deformation in the thigh region is beyond the scope of FFD. Local misalignment will affect the registration quality of entire image, which is not suitable for accurate dose mapping. However, with our proposed multi-phase (LFFD+FFD) registration strategy, the amount of misalignment in the thigh region has been reduced significantly.

**Fig 10:**
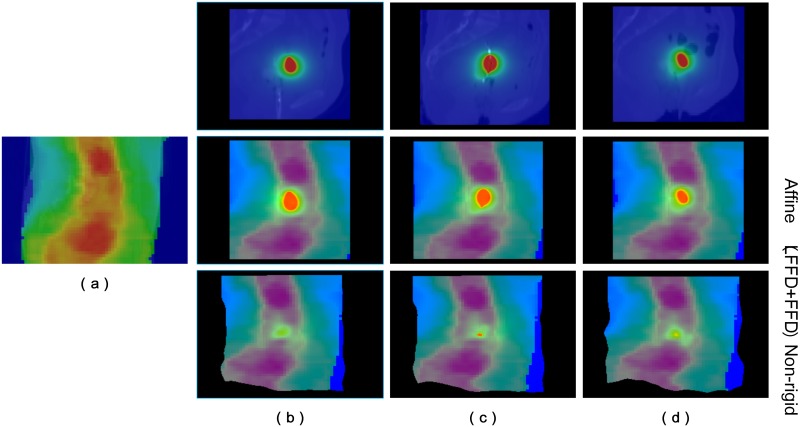
EBRT and HDR-BT dose accumulation in cervical cancer. (a) EBRT dose distribution used as a reference. The first, second, and third HDR-BT dose distributions and the accumulated dose distributions after affine and non-rigid (LFFD+FFD) registration are shown in columns (b)–(d).

After registration, all the HDR-BT images were mapped into the same referenced EBRT image space, and the corresponding transformations generated by registration were obtained. According to the scheme of dose accumulation shown in Fig 2, all the HDR-BT dose distributions were transformed into the reference EBRT dose distribution space according to the corresponding transformations, which are shown in Fig 10, taking three HDR-BT treatments and one EBRT treatment as examples.

**Fig 11:**
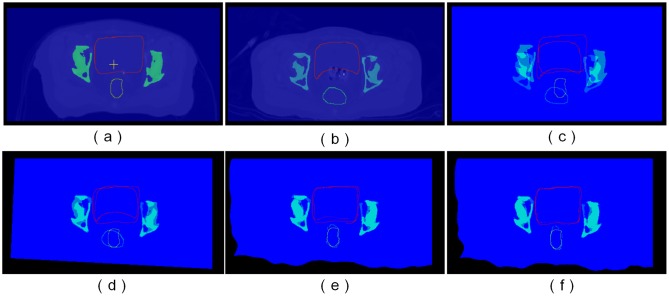
Validation of alignment of images by mapping contours of important organs. The contours of the pelvis, bladder, and rectum are delineated by an expert on both EBRT (a) and HDR-BT images (b). Alignment of both contours images without registration (c), and after affine (d), FFD (e), and LFFD+FFD registration (f).

It can be seen that after affine registration the accumulated dose distribution was roughly calculated by simply adding the dose distributions between EBRT and HDR-BT together. This was not the exact total dose received by different sites such as the tumor, bladder, rectum, and other organs during an entire course of EBRT and HDR-BT treatments. However, the multi-phase non-rigid method (LFFD+FFD), which takes the deformation of soft tissue into account, is more accurate for dose fusion and can be used to calculate dose accumulation. In our proposed approach, the validation of dose mapping was approximated by the validation of the accuracy of image registration. In this paper, we used the contours of the pelvis, bladder, and rectum as a reference, which were delineated by an expert in both EBRT and BT images as shown in Fig 11(a) and 11(b). Transformation generated by registration was applied to map the contours in the HDR-BT images onto the EBRT images. We also compared the results from different types of registration (affine, FFD, and LFFD+FFD) with a correlation coefficient (CC) to provide an indirect measure of registration quality. The corresponding registration results are shown in Fig 11(d)–11(f). For affine registration, a shift in the pelvis can be corrected easily, but this works poorly for soft tissue such as the bladder and rectum. However, FFD and LFFD+FFD methods can succeed in aligning these soft tissues with greater flexibility.

Table 1 summarizes the results for the quality of registration of EBRT and HDR-BT images in cervical cancer in terms of the CC for hybrid registration strategies, using initial control point spacing of 25, 20, and 15 mm respectively, in intensity-based FFD registration. The results for CT images with and without denoising are also compared. All registration methods improved the correlation between EBRT and HDR-BT images in comparison to no registration. However, FFD and LFFD+FFD performed better than the affine transformation model, and different control point spacing provided different registration results. The optimal initial control point spacing in FFD method was 20 mm, whereas in the LFFD+FFD method the performance was improved when the control point spacing decreased. The optimal initial control point spacing for intensity-based registration in the LFFD+FFD method was 15 mm. in these experiments. In all 11 sets of training data, LFFD+FFD registration performed better than FFD registration. Our proposed method can be more robust in dealing with large, local deformation in the thigh region in EBRT and HDR-BT images, which presents a challenge for conventional state-of-the-art registration methods. In addition, the results in Table 1 show that no matter what registration methods were used, the noise induced by the brachytherapy applicator reduced the correlation between the images seriously. In addition, image denoising is important for the registration of EBRT and HDR-BT images when normalize mutual information is chosen as a similarity metric.

**Table 1:** Comparison of the average registration errors for 11 sets of clinical data in terms of the CC for different registration strategies.

CT Image Without Denoising		CT Image With Denoising	
Registration	CC	Registration	CC
No registration	0.727±0.066	No registration	0.736±0.066
Affine	0.796±0.036	Affine	0.805±0.034
FFD (25mm)	0.973±0.015	FFD (25mm)	0.977±0.013
FFD (20mm)	0.974±0.017	FFD (20mm)	0.978±0.015
FFD (15mm)	0.971±0.025	FFD (15mm)	0.975±0.023
LFFD+FFD(25mm)	0.979±0.009	LFFD+FFD(25mm)	0.983±0.007
LFFD+FFD(20mm)	0.982±0.007	LFFD+FFD(20mm)	0.985±0.006
LFFD+FFD(15mm)	0.983±0.006	LFFD+FFD(15mm)	0.987±0.005

**Fig 12:**
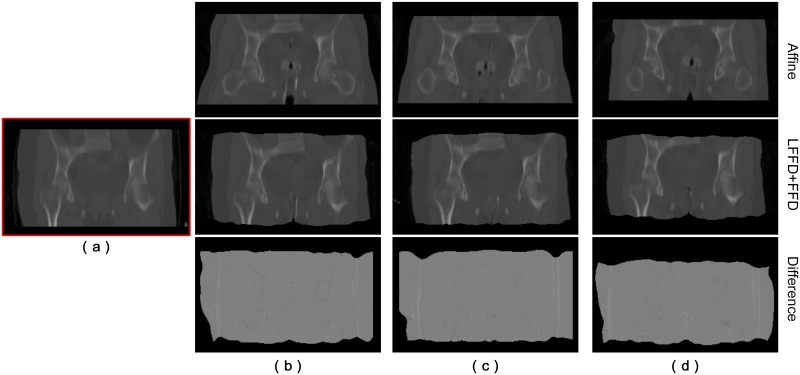
Mapping of three HDR-BT images onto a reference EBRT image using (LFFD+FFD) non-rigid registration. (a) EBRT image used as a reference. The first, second, and third HDR-BT images, registration result, and the corresponding difference image between EBRT and HDR-BT are shown in columns (b)–(d).

Using the same registration strategy (LFFD+FFD), we registered other HDR-BT images to the reference EBRT image throughout the entire course of treatment. Fig 12 shows the registration results and the corresponding difference images between EBRT and BT after (LFFD+FFD) non-rigid registration.

## 4. Discussion

The experimental results clearly show that complex image registration and dose fusion of EBRT and HDR-BT in cervical cancer can be effectively performed in FZUImageReg. For accurate dose assessment across EBRT and HDR-BT in cervical cancer, we found that it is not sufficient to simply add the dose distribution of each fraction together without taking deformations of soft tissue into account. Deformable image registration is a crucial technique for dose mapping, as shown in previous works [19–23]. However, the direct application of state-of-the-art image registration methods is not enough for accurate alignment of EBRT and HDR-BT CT images, owing to the large anatomical deformations between EBRT and HDR-BT induced by the brachytherapy applicator in the patient’s vagina. Local misalignment in the thigh region in EBRT and HDR-BT images compounds the difficulties of registration. We also showed that noise in the form of the uneven distribution of bright voxels induced by the brachytherapy applicator also significantly affected the accuracy of registration. As the accuracy of the assessment of dose accumulation significantly relies on the quality of registration, our proposed FZUImageReg system is able to deal with complex anatomical deformations in EBRT and HDR-BT images for cumulative dose assessment throughout the course of multiple treatments.

Although there are some commercially available registration software, the large deformation image in in cervical cancer therapy makes them extremely challenging for registration. It has been reported that six registrations failed out of 15 patients due to the “unreasonable” anatomical deformations between the EBRT and HDR-BT fractions [23]. Moreover, most software does not provide dose mapping functions directly. Rather than implementing a single registration method, FZUImageReg provides a wide collection of point-based and intensity–based registration methods. The modular design and user-friendly interface of our proposed system allow the user to quickly configure, test, monitor, and compare different registration methods for a specific application. Therefore, in our system, multi-phase non-rigid registration using LFFD can be easily designed and carried out for locally large deformation between EBRT and HDR-BT CT images, followed by intensity-based FFD. We can easily configure the parameters of the algorithm without writing any code in command line. Dose fusion and visualization functions are also integrated into the system. FZUImageReg can provide the entire pipeline of the process of dose fusion. Doctors and medical staff who are not familiar with image registration algorithms can easily complete complex registration tasks and determine radiotherapy dose accumulation via the user-friendly interface in our system.

However, the default parameter settings may not be optimal for other applications. For example, the optimal control point spacing in the registration of EBRT and HDR-BT images is 20 mm in the FFD method and 15 mm in the LFFD+FFD method, which may not be appropriate for other applications. Once the properties (spatial resolution, modality) of the images change, the parameters need to be tuned for optimal transformation. Evaluation of the accuracy of dose mapping is still a challenging task. On the basis of our approach, it could be approximated by evaluation of the quality of the registration of medical images. The evaluation of errors in deformable registration in our experiments was only based on CC, and also the overlap of manually defined organ contours.

Further work is needed to extend the functions of the proposed system and to improve the LFFD registration method presented in this paper, in order to minimize errors due to manual intervention. Some robust non-rigid transformation models will be integrated into the system, such as parameterized transformation models, which have previously been described [38] and non-parametric transformation models [39]. It is also necessary to speed up registration based on CUDA. In the field of radiation dose accumulation, we will study more patient data and conduct more clinical experiments, to further assess the accuracy of dose accumulation based on our proposed approach. In addition, the quality of visualization of the radiation dose distribution can also be improved in the system, to calculate the radiation dose in real time, and isodose lines will be provided to indicate the dose volume received by the tumor and organs at risk. This can provide a dosimetric reference for assessing the influence of radiotherapy to the local control rate and complication rate of cervical cancer.

## 5. Conclusions

In this paper, a framework of computer-aided dose fusion is proposed for use in the technology of image-guided intensity-modulated radiotherapy combined with 3D after-loading brachytherapy. To the best of our knowledge, FZUImageReg represent the first user-friendly medical image registration and dose fusion system. Several state-of-the-art registration methods are integrated into the proposed system, including rigid, affine, and non-rigid registration. A multi-phase non-rigid registration method using LFFD is proposed for locally large deformation between EBRT and HDR-BT CT images in cervical cancer, followed by intensity-based FFD. We have described how FZUImageReg can be used for the accurate registration of EBRT and HDR-BT CT images and3D dose accumulation over the entire course of image-guided radiation therapy. The experimental results clearly show that the proposed LFFD+FFD registration strategy is able to deal with the complex anatomical deformations in cervical cancer. In addition, our proposed system may be useful for further utilizing the advantages of the combination of EBRT and HDR-BT treatments and for facilitating personalized image-guide radiotherapy for cervical cancer patients.

## Supporting information

S1 File Medical image dataset. Original clinical data of cervical cancer patent without any identifying patent information.
(RAR)

S2 File FZUImageReg software. FZUImageReg software.
(RAR)
